# Live cell imaging of β-tubulin mRNA reveals spatiotemporal expression dynamics in the filamentous fungus *Aspergillus oryzae*

**DOI:** 10.1038/s41598-024-64531-5

**Published:** 2024-06-14

**Authors:** Keishu Kawatomi, Yuki Morita, Yoshinori Katakura, Kaoru Takegawa, Adokiye Berepiki, Yujiro Higuchi

**Affiliations:** 1 Department of Bioscience and Biotechnology, Faculty of Agriculture, Kyushu University, 744 Motooka, Fukuoka, 819-0395 Japan; 2 FUJIFILM Diosynth Biotechnologies, Billingham, TS23 1LH UK

**Keywords:** *Aspergillus oryzae*, Filamentous fungi, Microtubule, Mitosis, mRNA, Tubulin, Cell biology, Microbiology

## Abstract

In filamentous fungi, microtubules are important for polar growth and morphological maintenance and serve as rails for intracellular trafficking. The molecular mechanisms associated with microtubules have been analyzed. However, little is known about when and where tubulin, a component of microtubules, is biosynthesized in multinuclear and multicellular filamentous fungi. In this study, we visualized microtubules based on the enhanced green fluorescence protein (EGFP)-labeled α-tubulin and β-tubulin mRNA tagged by the EGFP-mediated MS2 system in living yellow *Koji* mold *Aspergillus oryzae* cells in order to understand the spatiotemporal production mechanism of tubulin. We found that mRNA of *btuA*, encoding for β-tubulin, localized at dot-like structures through the apical, middle and basal regions of the hyphal cells. In addition, some *btuA* mRNA dots showed microtubule-dependent motor protein-like dynamics in the cells. Furthermore, it was found that *btuA* mRNA dots were decreased in the cytoplasm just before mitosis but increased immediately after mitosis, followed by a gradual decrease. In summary, the localization and abundance of β-tubulin mRNA is spatiotemporally regulated in living *A. oryzae* hyphal cells.

## Introduction

Microtubules are one of the major cytoskeletons in eukaryotes and are tubular structures with α-tubulin and β-tubulin dimers as the basic building blocks. The polymerization and depolymerization of α/β-tubulin heterodimers cause microtubules to elongate and shorten repeatedly, forming a network in the cytoplasm. Microtubules are necessary for polar growth and morphological maintenance of cells, and also act as rails for intracellular transport. Moreover, they form spindles during cell division and play an important role in the even distribution of chromosomes^1,2^. Dynamics caused by repeated polymerization and depolymerization of α/β-tubulin heterodimers are important for the functions of microtubules. Microtubule elongation increases linearly in dependence on cytoplasmic tubulin concentration^3^. In addition, phase separation of Tip-Interacting Proteins (+TIPs), such as End-Binding proteins (EBs), concentrates tubulin and promotes microtubule formation^2,4, 5^.

Microtubules in filamentous fungi have been well studied in *Aspergillus nidulans*^6^. In particular, the genes for α-tubulin and β-tubulin were first identified in *A. nidulans*, and γ-tubulin, a third tubulin important for microtubule formation, was also found in *A. nidulans*^7–9^. In fungi, γ-tubulin localizes to the spindle pole body (SPB), is important for the formation of microtubule elongation nuclei and serves as a scaffold for α/β-tubulin heterodimer polymerization^10,11^. Furthermore, in *A. nidulans*, γ-tubulin has also been reported to localize to septal microtubule-organizing centers (sMTOCs) and to elongate microtubules from sMTOCs^12,13^. Microtubules extending into the cytoplasm from SPBs and sMTOCs form a highly complex network within the elongated cells of filamentous fungi and serve as transport tracks for secretory vesicles, endosomes and other organelles^14–16^.

Messenger RNA (mRNA) localization in cells is important for the spatiotemporal regulation of gene expression and is a widely conserved biological process from prokaryotes to eukaryotes^17–21^. Localization of mRNA to specific sites in the cell is important to ensure that individual proteins are in the right place and is a necessary physiological function to prevent mislocalization of proteins^22^. In addition, mRNAs localized to specific sites can be translated multiple times, saving the energy needed to transport protein^23^. Furthermore, mRNA localization control is thought to be important for local protein synthesis^24^, and especially in multicellular organisms, disruption of proper mRNA localization has been reported to cause significant developmental and growth abnormalities^25–27^. The molecular mechanisms of mRNA localization are being analyzed with a focus on its physiological significance, but many aspects remain unknown.

The yellow *Koji* mold *Aspergillus oryzae* is a filamentous fungus widely used in the fermentation and brewing industries and is capable of secreting and producing large amounts of various useful enzymes safely, so studies on membrane traffic including the secretory pathway are well established^28,29^. Such studies have shown that microtubules are important in *A. oryzae* as rails for intracellular material transport^30^. Hydrolytic enzymes, such as amylases, which are abundantly produced in *A. oryzae*, are transported to secretory sites such as hyphal tips and septa in the presence of microtubules, and proper formation of microtubules is important for *A. oryzae* to efficiently produce secretory proteins out of the cell^31^. In *A. oryzae*, the localization of each mRNA and translated protein, including α-amylase and actin, was analyzed by single-molecule fluorescence in situ hybridization^32,33^. Furthermore, mRNA for glucoamylase (GlaA), one of the major secretory proteins in *A. oryzae*, was analyzed by the MS2 system, which enables mRNA to be visualized in living cells, revealing that *glaA* mRNA is transcribed from the nucleus near the hyphal tip and septum, the sites of secretion^34^. However, filamentous fungi, including *A. oryzae*, are multinuclear and multicellular, and the mechanisms that regulate the spatiotemporal expression of proteins that function intracellularly, such as tubulin, are largely unknown. Therefore, in this study, we analyzed the spatiotemporal dynamics of tubulin mRNA, which is a component of microtubules.

## Results

### Microtubule dynamics in *A. oryzae* hyphal cells

**Figure 1:**
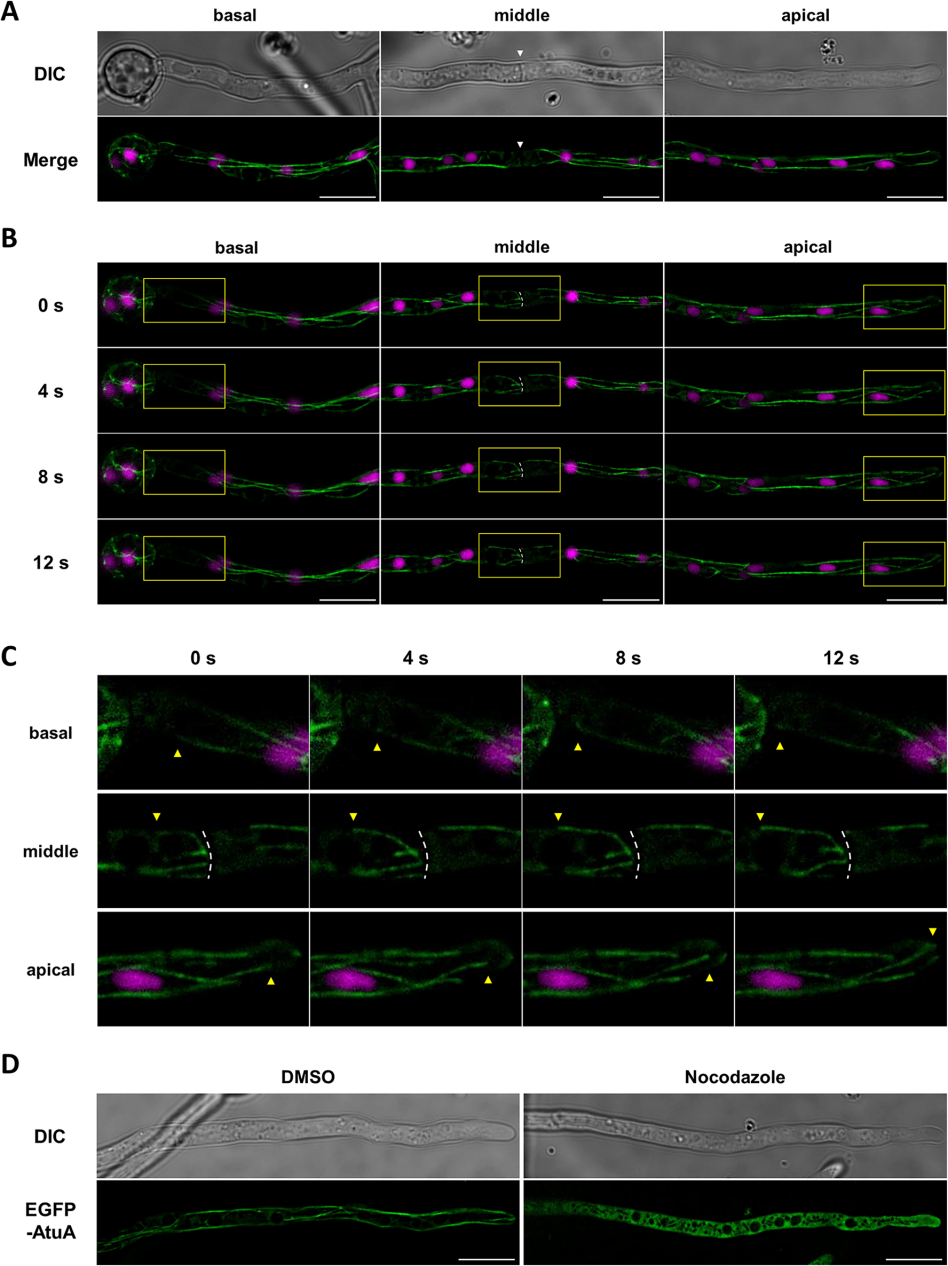
Dynamics of microtubules in *A. oryzae* hyphal cells. (**A**) Colocalization of microtubules (EGFP-AtuA, green) and nuclei (mCherry-NLS, magenta). White arrowheads point to septa. (**B**) Time-lapse images of (**A**) were taken at 2 s intervals, and images at 0 s, 4 s, 8 s and 12 s are shown. White dot lines indicate septa. (**C**) Enlarged time-lapse images of the yellow frames in (**B**). Yellow arrowheads indicate elongating microtubule ends at each region. (**D**) Localization of microtubules in cells treated with DMSO or nocodazole. Scale bars, 10 µm.

In filamentous fungal cells, microtubules have been shown to exhibit dynamics in the cytoplasm, but in *A. oryzae* they have not been analyzed in detail. Thus, we fused enhanced green fluorescence protein (EGFP) to the N-terminus of AtuA (AO090005000840), an α-tubulin in *A. oryzae*, and also labeled the nucleus with red fluorescence protein mCherry-tagged nuclear localization signal (NLS). EGFP-AtuA was visualized as filaments in the cytoplasm from the basal to the tip regions (Fig. 1A). Its localization pattern is similar to its homolog protein previously seen in *A. nidulans*, suggesting similar microtubule formation mechanisms and functions^35^.

To further investigate the dynamics of elongated microtubules in the cytoplasm, time-lapse analysis at 2-s intervals revealed large fluctuations of microtubules throughout the cytoplasm and elongation of filament structures regardless of the hyphal regions (Fig. 1B, C, Supplementary videos S1–3). Focusing on the middle part of the hyphal cells, studies in *A. nidulans* have reported the presence of sMTOCs as microtubule-forming centers localized at the septum and microtubules extending from sMTOCs to the cytoplasm^13^. Furthermore, in *Neurospora crassa*, microtubules have been observed penetrating the septal pore at the center of the septum^36^. In *A. oryzae*, multiple microtubule filaments were present near the septum, and they elongated starting from the septum, suggesting the presence of sMTOC. EGFP-AtuA also diffused into the cytoplasm upon nocodazole treatment, an inhibitor of microtubule polymerization, indicating that EGFP-AtuA indeed constitutes microtubules (Fig. 1D).

### Visualization of *btuA* mRNA by the MS2 system in living *A. oryzae* cells

**Figure 2:**
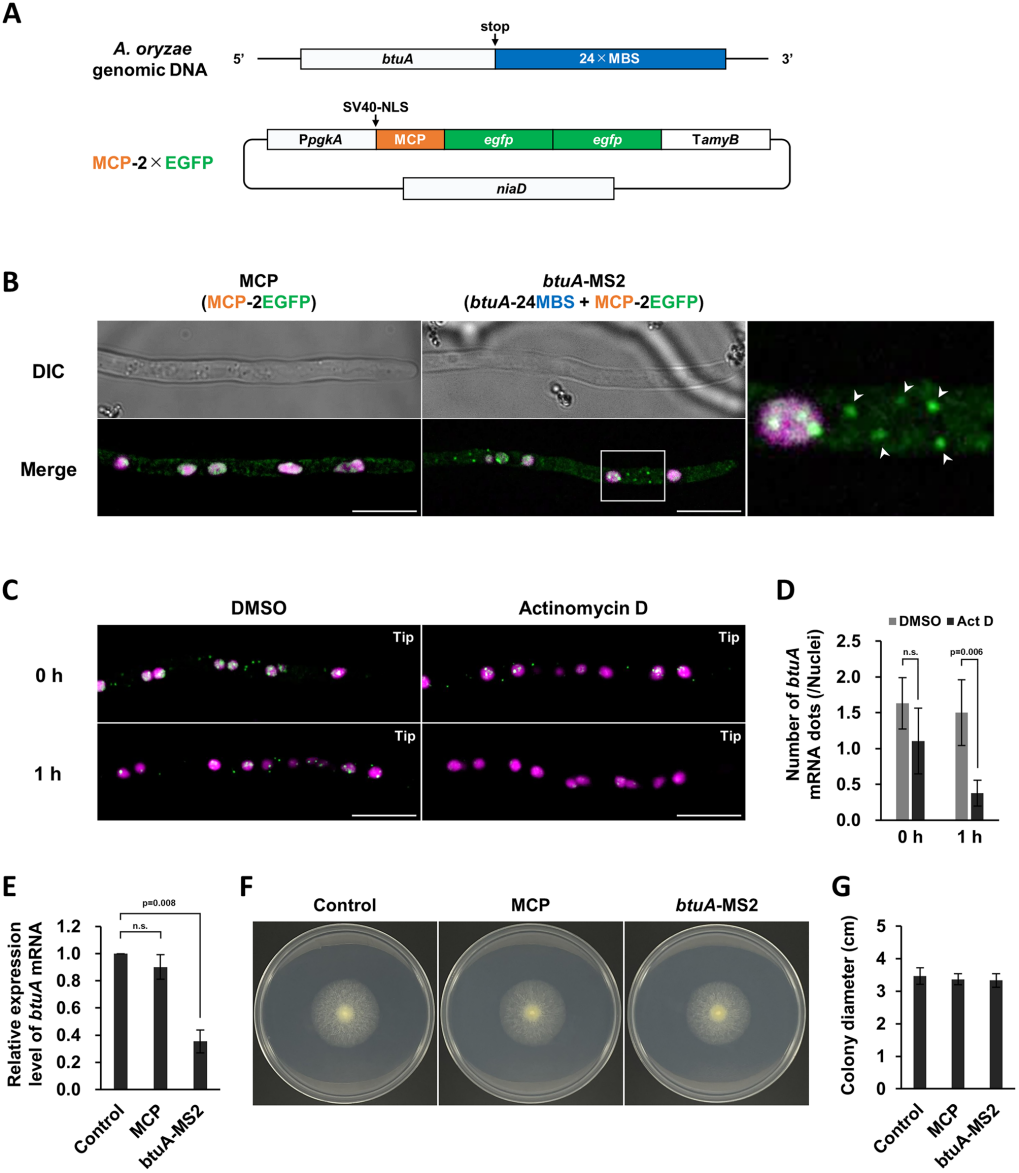
Visualization of *butA* mRNA by the MS2 system in *A. oryzae* living cells. (**A**) Scheme of strategy for visualization of *btuA* mRNA by the MS2 system. 24×MBS was inserted into the 3′UTR of endogenous *btuA* gene and NLS-fused MCP-2×EGFP was co-expressed. (**B**) Visualization of MCP-2×EGFP or *btuA* mRNA (green) and nuclei (magenta) in the apical cells. In the enlarged image on the right, white arrowheads point to *btuA* mRNAs localized in the cytoplasm. Scale bars, 10 µm. (**C**) Visualization of *btuA* mRNAs (green) and nuclei (magenta) in cells treated with DMSO or actinomycin D. Scale bars, 10 µm. (**D**) The number of *btuA* mRNA dots localized in the cytoplasm in each culture condition. Error bars indicate standard deviation of the mean (n = 5). Student’s t-test, no significant difference at 0 h (*p* = 0.1075). (**E**) Relative expression level of *btuA* mRNA. Error bars indicate standard deviation of the mean (n = 3). Student’s t-test, no significant difference between the control and MCP strains (*p* = 0.2604). (**F**) Growth tests of the control, MCP and *btuA*-MS2 strains. (**G**) Diameter of colony in each strain. Error bars indicate standard deviation of the mean (n = 3).

There have been no reports of live cell imaging of mRNA encoding tubulin, a component of microtubules, in any cell type. Therefore, in order to analyze tubulin mRNA and microtubules simultaneously in *A. oryzae*, we decided to analyze microtubules with α-tubulin AtuA as described above and visualize β-tubulin mRNA. The MS2 system is often used to visualize mRNA in living cells, and in fact, mRNA for *glaA*, which encodes glucoamylase, a secreted enzyme, has been visualized and analyzed in *A. oryzae*^17,34^. We introduced 24 copies of the aptamer MS2-binding sites (MBS) downstream of the endogenous *btuA* (AO090009000281) encoding β-tubulin and generated a *btuA*-MS2 strain that expresses two copies of EGFP fused to MS2-coat protein (MCP), which binds specifically to MBS (Fig. 2A). To eliminate background signals when analyzing cytoplasmic *btuA* mRNA, NLS was added to the N-terminus of MCP-2×EGFP so that NLS-MCP-2×EGFP localizes to the nucleus when *btuA* mRNA is not expressed (Fig. 2A). When mCherry-NLS was also expressed to label the nucleus, *btuA* mRNA showed dot-like fluorescence in the cytoplasm in the *btuA*-MS2 strain, whereas NLS-MCP-2×EGFP was only localized to the nucleus in the strain without MBS (Fig. 2B). The microscopic analysis system used in this study did not detect the NLS-MCP-2×EGFP signal in the cytoplasm. In addition, although Z-stack was not used, the THUNDER Imager Live Cell system used in this study succeeded in obtaining clear images by computational clearing. Cytoplasmic dot fluorescence was not observed after treatment with the transcriptional inhibitor actinomycin D (Fig. 2C, D). These results suggest that the cytoplasmic dot fluorescence observed in the *btuA*-MS2 strain is derived from *btuA* mRNA.

Analysis of *btuA* expression by quantitative reverse transcription PCR (qRT-PCR) showed that the amount of *btuA* mRNA in the *btuA*-MS2 strain was approximately 65% lower than in the control strain (Fig. 2E). In general, it has been reported that the addition of MBS sequences in the MS2 system reduces mRNA stability^37^, suggesting that about two-thirds of the *btuA* mRNA was degraded in the *btuA*-MS2 strain. However, comparative growth analysis of the *btuA*-MS2 strain revealed that the reduction in *btuA* mRNA expression did not have a marked effect on growth (Fig. 2F, G), so we decided to use the *btuA*-MS2 strain for future analysis.

### *btuA *mRNA exists through the *A. oryzae* hyphal cells

**Figure 3:**
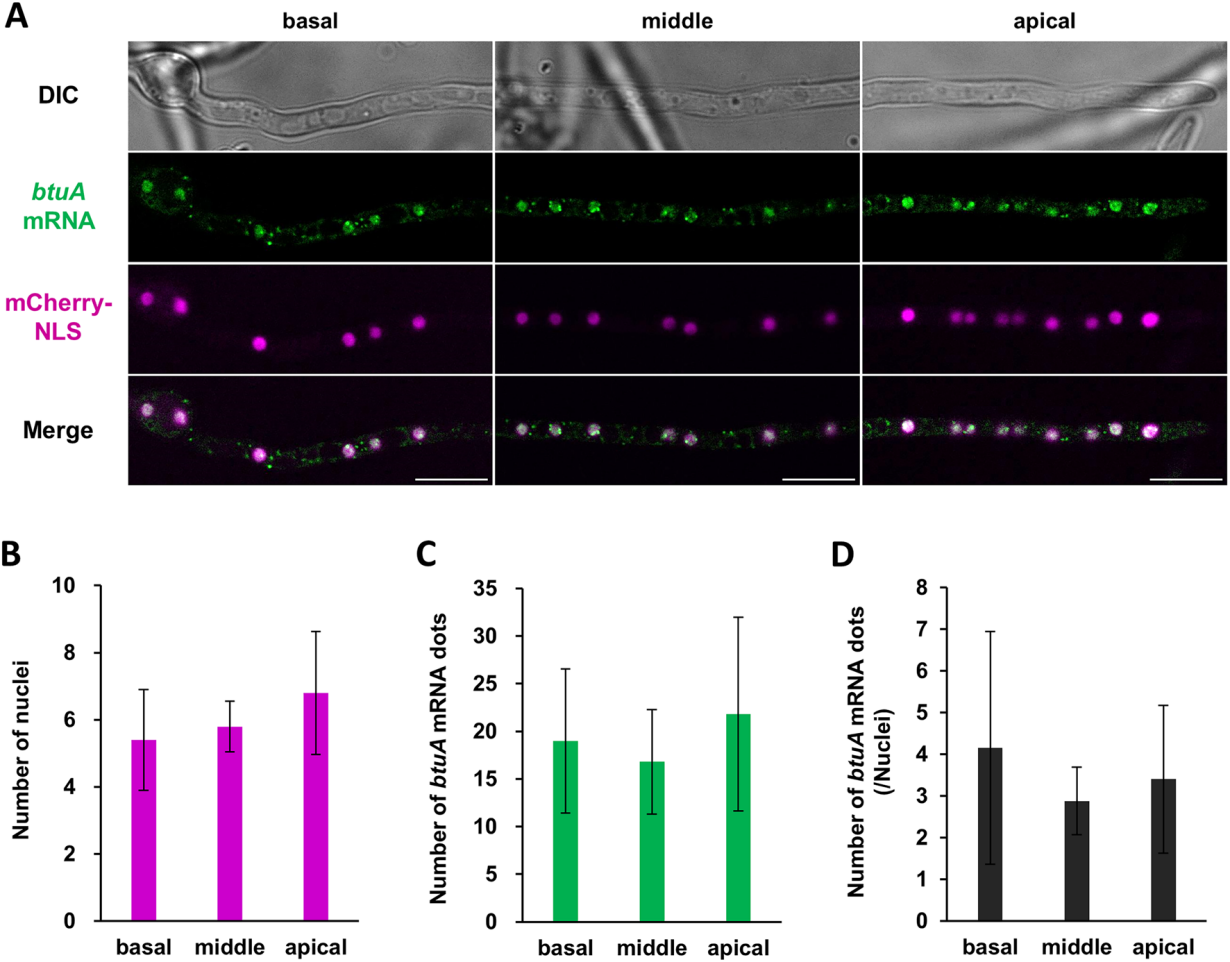
Distribution of *btuA* mRNA in *A. oryzae* hyphal cells. (**A**) Localization of *btuA* mRNA (green) and nuclei (magenta) in each hyphal area. Scale bars, 10 µm. (**B**–**D**) The number of nuclei (**B**), *btuA* mRNA dots (**C**) and *btuA* mRNA dots/nuclei (**D**) in each hyphal area. Tukey–Kramer test does not show significant difference among hyphal regions. Error bars indicate standard deviation of the mean (n = 5).

To determine how *btuA* mRNA is distributed in *A. oryzae* hyphal cells, we performed a localization analysis of *btuA* mRNA in each hyphal region. We analyzed the *btuA*-MS2 strain by introducing the mCherry-NLS construct into the *btuA*-MS2 strain to label the nuclei (Fig. 3A). First, we analyzed whether there was a gap in the number of nuclei in each hyphal region. The results showed that there was no significant difference in the number of nuclei in each hyphal region, and the number of nuclei was almost the same (Fig. 3B). Then, we quantified the number of dots shown by *btuA* mRNA and found that *btuA* mRNA was almost uniformly localized in the cytoplasm, regardless of the hyphal regions (Fig. 3C). The number of *btuA* mRNA dots per number of nuclei also did not differ among hyphal regions (Fig. 3D). These results indicate that *btuA* mRNA is uniformly expressed and present regardless of the hyphal regions.

### The dynamics of *btuA* mRNA are dependent on microtubules

**Figure 4:**
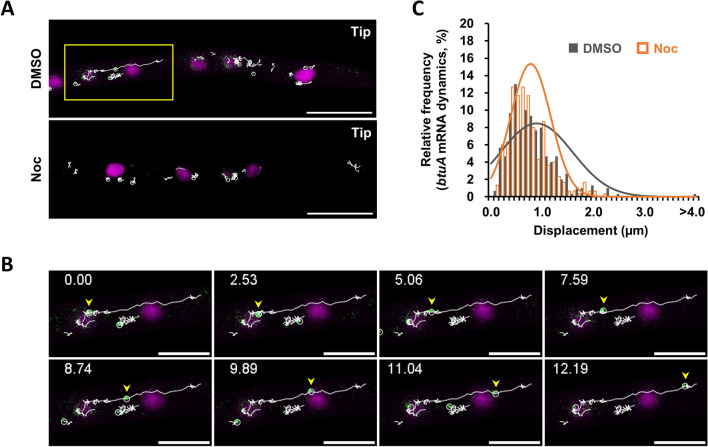
The dynamics of *btuA* mRNA are dependent on microtubules. (**A**) Tracking analysis for *btuA* mRNAs in cytoplasm treated with DMSO or nocodazole. Scale bars, 10 µm. (**B**) Enlarged time-lapse images of the yellow frame in (**A**). Yellow arrowheads indicate the linear motility of *btuA* mRNA likely via a motor protein. Numbers in sec. Scale bars, 5 µm. (**C**) Histograms showing the displacement of *btuA* mRNAs in each culture condition. Non-parametric Mann–Whitney U-test shows a significant difference, *p* = 0.0152 (DMSO: n = 300, median = 0.786 µm, max = 10.5 µm, min = 0.139 µm; Noc: n = 300, median = 0.696, max = 2.28 µm, min = 0.156 µm).

It has been shown that in *A. oryzae*, a portion of the mRNA for *glaA*, which encodes the secretory protein glucoamylase, migrates long distances on microtubules in a kinesin motor-dependent manner^34^. Therefore, nocodazole treatment, a microtubule polymerization inhibitor, was first applied to analyze the microtubule dependence of *btuA* mRNA dynamics. The results showed that in the control DMSO treatment, *btuA* mRNA exhibited motor protein-like kinetics with linear long-distance movement after migration from the nucleus to the cytoplasm (Fig. 4A, B, Supplementary videos S4, S5). Assuming that such long-distance dynamics are microtubule-dependent, we quantified the linear migration distance of *btuA* mRNA monitored by tracking analysis and showed that nocodazole treatment shortened the displacement of *btuA* mRNA (Fig. 4C). Reviewing the data on 300 *btuA* mRNA movements, as shown in Fig. 4C, long-range movement as in Fig. 4B was less common, and long-range and bi-directional movement was not observed. These results indicate that *btuA* mRNA, like *glaA* mRNA, exhibits microtubule-dependent long-range dynamics.

### The abundance of *btuA* mRNA in the cytoplasm is dependent on cell cycle

**Figure 5:**
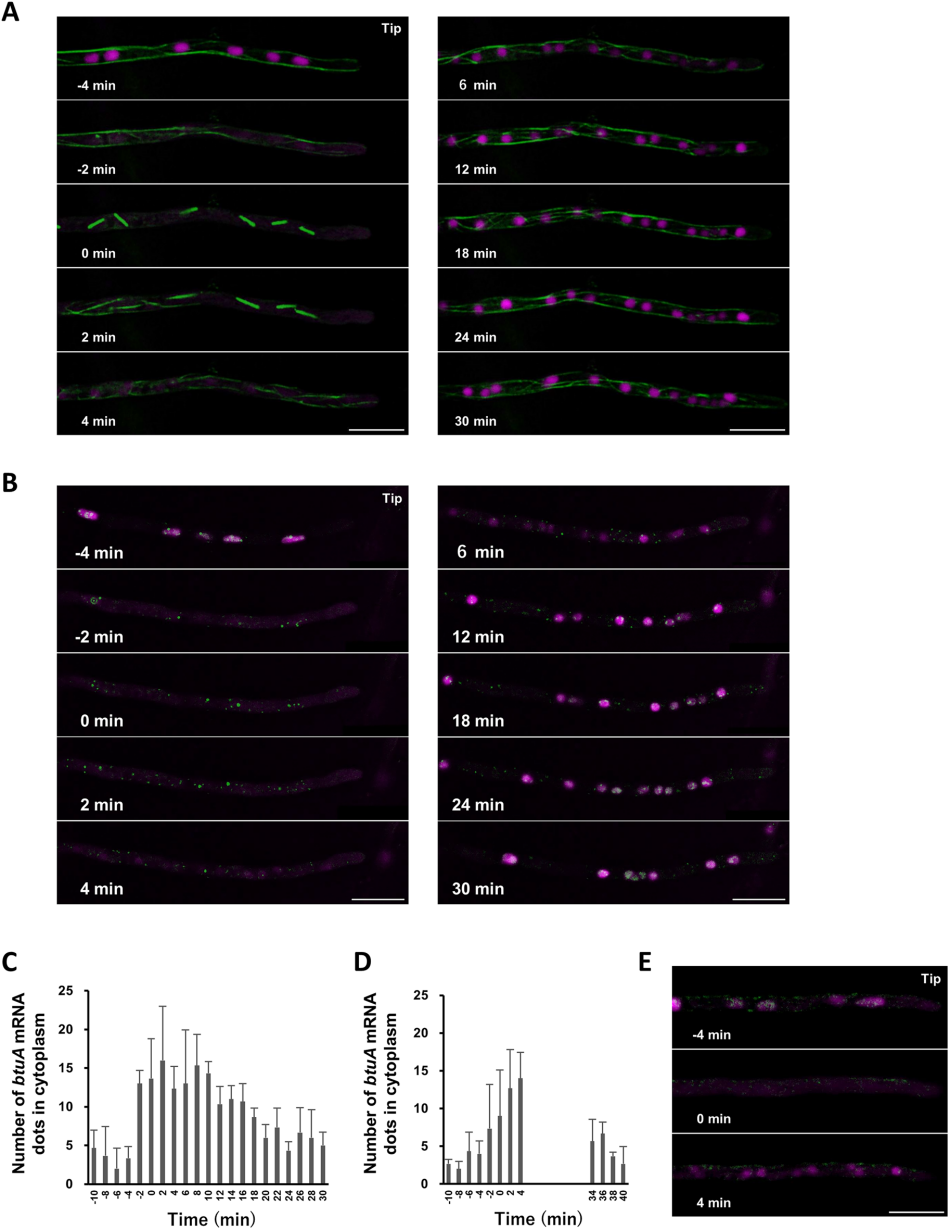
Expression dynamics of *btuA* mRNA during mitosis. (**A**) Time-lapse images of microtubules (green) and nuclei (magenta) in the cell cycle. Microtubules form spindles at all nuclei of the apical region, set at 0 min. Scale bars, 10 µm. (**B**) Time-lapse images of *btuA* mRNA dynamics in the cell cycle. (**C** and **D**) The number of *btuA* mRNA dots were counted in the cytoplasm of the region of 50 µm from the hyphal tip. In (**D**), a 30 min interval was given from 4 to 34 min. Error bars indicate standard deviation of the mean (n = 3). (**E**) Time-lapse images of NLS-MCP-2×EGFP (green) and nuclei (magenta) under mitosis. Scale bar, 10 µm.

Microtubules dynamically change their localization in a cell cycle-dependent manner. Time-lapse analysis of *A. oryzae* strains labeled with microtubules and nuclei in mitosis clearly showed that microtubules formed spindles at the timing of nuclear division, followed by the re-formation of doubled nuclei and cytoplasmic microtubules within 10 min (Fig. 5A). Then, we analyzed *btuA* mRNA in mitosis and found that the number of *btuA* mRNA dots in the cytoplasm decreased just before mitosis, reached a maximum at the time of mitosis, and then slowly decreased (Fig. 5B, C). To confirm that the decrease in *btuA* mRNA in the cytoplasm after mitosis was not due to fading of the fluorescent protein, because of the long time-lapse analysis, we observed a 30-min interval immediately after mitosis and obtained similar results (Fig. 5D). To confirm whether the observed cytoplasmic dot fluorescence was actually indicative of *btuA* mRNA, we observed NLS-MCP-2xEGFP in the strain lacking MBS. During mitosis, NLS-MCP-2xEGFP diffused into the cytoplasm and showed no dot-like localization, but upon return to interphase it again showed nuclear localization, indicating that the dot fluorescence observed in the *btuA*-MS2 strain was derived from *btuA* mRNA (Fig. 5E). These results demonstrate that *btuA* mRNA changes its amount in the cytoplasm in response to the cell cycle.

## Discussion

To our best knowledge, tubulin mRNA has never been reported to be visualized in living cells of any species. Therefore, in this study, we attempted to visualize *A. oryzae* live cells and found that the MS2 system enables visualization of *btuA* mRNA, which encodes β-tubulin, and that spatiotemporal expression dynamics exist.

*btuA* mRNA was shown to be uniformly expressed from the basal to the tip regions and localized throughout the cytoplasm of hyphal cells. It is conceivable that the *btuA* mRNA found in the nucleus could be present in the nucleus or also localized to the SPB. It has been analyzed in *A. nidulans* with ApsB as a marker for SPB^38^, and future co-localization analysis with the *A. oryzae* ortholog AoApsB (AO090020000040) is possible.

The quantitative data on the number of nuclei and cytoplasmic mRNA suggested that the same amount of *btuA* mRNA was expressed per nucleus at all sites in the hyphal cells. This mode of expression of *btuA* mRNA differed from the finding that *glaA* mRNA, encoding the secretory protein glucoamylase, is transcribed predominantly in nuclei near the hyphal tip and septum^34^. In filamentous fungi, secretory proteins are secreted from the hyphal tips and septa^31^, so it makes sense that *glaA* mRNA is preferentially expressed in nuclei near such sites. On the other hand, if we consider that microtubules exhibit dynamics throughout the mycelial cell and are repeatedly turned over at the protein level, this is consistent with the fact that *btuA*-encoded β-tubulin mRNA is expressed in hyphal regions without being restricted to them. Microtubules serve as rails for hyphal cell morphology maintenance and secretory vesicle and early endosomal transport^30^. This is consistent with the need for a constant and extensive supply of tubulin mRNA for microtubule function not limited to specific hyphal regions. These results suggest that proteins secreted extracellularly and those functioning intracellularly are spatially regulated by different mechanisms.

The kinetic analysis of *btuA* mRNA suggested the involvement of microtubules in its localization. Indeed, in vitro analysis of mouse β-tubulin mRNA reported that β-tubulin mRNA is regulated by RNA-binding protein (RBP)-mediated transport by kinesin motor proteins^39^. Thus, it is expected that in *A. oryzae*, *btuA* mRNA also undergoes kinesin motor-dependent transport regulation. To further investigate the association between β-tubulin mRNA localization and microtubules in *A. oryzae*, we need to attempt to identify RBPs that interact with β-tubulin mRNA and RBP binding sites on mRNA. Many proteins are involved in microtubule regulation as microtubule-interacting proteins, and it is necessary to analyze the extent to which β-tubulin mRNA is also regulated by RBPs in the future.

In mitosis, microtubules dynamically change their localization. Time-lapse analysis focusing on the cell cycle revealed that in *A. oryzae*, spindle formation occurs simultaneously in multiple nuclei, and that the disappearance and formation of cytoplasmic microtubules are carried out in a short period of about 10 min before and after the spindle formation. This phenomenon has also been observed in *A. nidulans* and is reported to proceed at a rate comparable to the mitosis observed here in *A. oryzae*^40^. We found that *btuA* mRNA in the cytoplasm increased rapidly during mitosis, followed by a gradual decrease. It is likely that translation occurs in response to the increasing amount of *btuA* mRNA, resulting in the de novo synthesis of β-tubulin. However, the molecular regulation mechanism and physiological significance of this phenomenon are currently unknown. The SunTag method is a technique to visualize translated mRNA^41–45^, and the degree of *btuA* mRNA translation should also be analyzed in the future. In addition, further work should attempt to clarify how tubulin mRNA localization throughout the cytoplasm and cell cycle-dependent expression changes revealed by this study are related to tubulin production and microtubule formation.

## Methods

### Culture media

Czapek-Dox (CD) (0.3% NaNO_3_, 0.2% KCl, 0.1% KH_2_PO_4_, 0.05% MgSO_4_·7H_2_O, 0.002% FeSO_4_·7H_2_O and 2% glucose, pH 5.5), CD supplemented with 0.0015% methionine (CDm) and CD or CDm supplemented with 0.1% of uracil and trace amount of uridine (CDUU, CDmUU) were used for culture media to fit with each strain auxotrophy. To drop off the *pyrG* selection marker and Cre expression cassette by the Cre/*loxP* system, each strain was cultured on a CDmUU plate with the carbon source replaced by 2% xylose.

### Plasmid and strain construction

The *A. oryzae* strains used in this study are listed in Table 1. Genomic DNA of the wild-type *A. oryzae* strain RIB40 was used as the template for the DNA cloning. For visualization of microtubules, the plasmid pgPaEGAtuA^34^ for expression of EGFP-AtuA was transformed into the NSlD1 strain to create the GaA1 strain. To further visualize nuclei, the plasmid pgPtmCN^34^ expressing mCherry-NLS was introduced into the GaA1 strain to create the GaA_CN1 strain.

**Table 1:** Strains used in this study.

Strain	Genotype	References
RIB40	Wild-type	
NSPlD1	*niaD*^*−*^* sC*^*−*^* adeA*^*−*^ Δ*argB*::*adeA*^*-*^ Δ*ligD*::*argB* Δ*pyrG*::*adeA*	^50^
NSPlDN1	*niaD*^*−*^* niaD sC*^*−*^* adeA*^*−*^ Δ*argB*::*adeA*^*−*^ Δ*ligD*::*argB* Δ*pyrG*::*adeA*	This study
NSlD1	*niaD*^*−*^* sC*^*−*^* adeA*^*−*^ Δ*argB*::*adeA*^*−*^ Δ*ligD*::*argB* Δ*pyrG*::*adeA pyrG*	^50^
NSlDS1	*niaD*^*−*^* sC*^*−*^* AosC adeA*^*−*^ Δ*argB*::*adeA*^*−*^ Δ*ligD*::*argB* Δ*pyrG*::*adeA pyrG*	^46^
GaA1	*niaD*^*−*^ (P*atuA-egfp-atuA niaD*) *sC*^*−*^* AosC adeA*^*−*^ Δ*argB*::*adeA*^*−*^ Δ*ligD*::*argB* Δ*pyrG*::*adeA pyrG*	This study
GaA_CN1	*niaD*^*−*^ (P*atuA-egfp-atuA niaD*) *sC*^*−*^ (P*Aotps1-mcherry-nls AosC*) *adeA*^*−*^ Δ*argB*::*adeA*^-^ Δ*ligD*::*argB* Δ*pyrG*::*adeA pyrG*	This study
MCPG2	*niaD*^*−*^ (P*pgkA-nls-mcp-2*×*egfp niaD*) *sC*^*−*^* adeA*^*−*^ Δ*argB*::*adeA*^*−*^ Δ*ligD*::*argB* Δ*pyrG*::*adeA*	This study
*btuA*-MS2	*niaD*^*−*^ (P*pgkA-nls-mcp-2*×*egfp niaD*) *sC*^*−*^* adeA*^*−*^ Δ*argB*::*adeA*^*−*^ Δ*ligD*::*argB* Δ*pyrG*::*adeA btuA-24*×*mbs*::*pyrG* Δ*pyrG*	This study
*btuA*-MS2_CaA1	*niaD*^*−*^ (P*pgkA-nls-mcp-2*×*egfp niaD*) *sC*^*−*^ (P*atuA-mcherry-atuA AosC*) *adeA*^*−*^ Δ*argB*::*adeA*^*−*^ Δ*ligD*::*argB* Δ*pyrG*::*adeA btuA-24*×*mbs*::*pyrG* Δ*pyrG*	This study
*btuA*-MS2_CN1	*niaD*^*−*^ (P*pgkA-nls-mcp-2*×*egfp niaD*) *sC*^*−*^ (P*Aotps1-mcherry-nls AosC*) *adeA*^*−*^ Δ*argB*::*adeA*^*−*^ Δ*ligD*::*argB* Δ*pyrG*::*adeA btuA-24*×*mbs*::*pyrG* Δ*pyrG*	This study

For *btuA* mRNA visualization, the plasmid pgPpNM2G^34^ expressing NLS-MCP-2×EGFP was first transformed into the NSPlD1 strain to create the MCPG2 strain. As a control strain, the NSPlDN1 strain was also generated by transforming only the *niaD* marker into the NSPlD1 strain. The MBS-containing vector sequences were then amplified by inverse PCR using PrimeSTAR MAX DNA polymerase (Takara) from the glaA-MBS cassette^34^ and primers YM115 (5′-CAGAGCCCCCTGGCAATCGC-3′) and YM122 (5′-GCGGCCGCGTTAACCGCTCA-3′), and ligated with approximately 1 kb, excluding the stop codon, of the *btuA* ORF amplified by PCR using PrimeSTAR GXL DNA polymerase (Takara) and primers KKT43 (5′-GGTTAACGCGGCCGCGGAACTCTTTTGATCTCGAAGAT-3′) and KKT44 (5′-TGCCAGGGGGCTCTGCTACTGCTCCTCGATCTCCT-3′). In this case, a plasmid with a 24×MBS of approximately 1.6 kb was selected. The vector sequences containing 24×MBS were then amplified by inverse PCR with primers YM116 (5′-TACCGTTCGTATAATGTATGCTATACGAAGTTATC-3′) and YM130 (5′-GCGGCCGCTATGGTGCACTC-3′), and ligated with approximately 1 kb of *btuA* 3′-UTR amplified with primers KKT45 (5′-ATTATACGAACGGTATTATCTCGTCACTATTCGCGG-3′) and KKT46 (5′-ACCATAGCGGCCGCGACACGGCCATAGTGCTATAC-3′) to produce the btuA-24×MBS cassette. The btuA-24×MBS was digested with NotI and transformed into the MCPG2 strain. Then, the *pyrG* marker was removed by the Cre/*loxP* system to produce the *btuA*-MS2 strain.

To visualize microtubules and nuclei with red fluorescence simultaneously with *btuA* mRNA, the *btuA*-MS2 strain was transfected with either pgPamCAtuA expressing mCherry-AtuA or pgPtmCN expressing mCherry-NLS to create the btuA-MS2_CaA1 or btuA-MS2_CN1 strains, respectively.

### Quantitative RT-PCR analysis

Quantitative reverse transcription PCR (qRT-PCR) analysis was performed as described previously^46^. The NSPlDN1, MCPG2 and *btuA*-MS2 strains were inoculated with 1.0 × 10^5^ conidia in 100 ml of CDmUU medium and cultured at 30 °C, 20 h, 150 rpm. Total RNA was then extracted from each mycelium according to the instructions of the RNeasy® Plant Mini Kit (Qiagen), and cDNA was synthesized using the ReverTra Ace qPCR RT Master Mix (Toyobo). Thermal Cycler Dice Real Time System TP-800 instrument (Takara) and Thunderbird SYBR qPCR Mix (Toyobo) were used for qRT-PCR analysis of each cDNA. Expression levels of *btuA* mRNA were measured using primers YHK297 (5′- TTCTTCATGGTGGGCTTTGCAC-3′) and YHK298 (5′-AAGCGGCCGTTGTGGAAATTAG-3′). Expression of *gpdA* as normalization was measured using primers YHK196 (5′-CGTCGAGTCCACTGGTGTCTT-3′) and YHK197 (5′-TTGTTGACACCCATAACGAACATGG-3′)^47^.

### Fluorescence microscopy

Two hundred µL of medium sterilized with 0.2 µm filter was added to a polylysine-coated glass bottom dish and inoculated with 5.0 × 10^4^ or 1.0 × 10^5^ conidia of each strain and cultured for 20 h at 30 °C. To inhibit transcription, Actinomycin D (Act D, stock concentration of 100 mg/µl in DMSO, FUJIFILM Wako) was used.

Live cell imaging was performed using THUNDER Imager Live Cell (Leica microsystems), equipped with an HCX PL APO 100×/1.40–0.70 OIL objective lens, a K8 Scientific CMOS camera, a DFT51010 filter (Leica: LED, DAPI_FITC_TXRED_Cy5-390/475/555/635; Excitation Range, DAPI_FITC_TXRED_Cy5-375-407/462-496/542-566/622-654; Emission Range, DAPI_FITC_TXRED_Cy5-420-450/506-532/578-610/666-724; Dichroic, DAPI_FITC_TXRED_Cy5-415/490/ 572/660) and a CYR71010 filter (Leica: LED, CFP_YFP_RFP_NIR-440/510/575/730; Excitation Range, CFP_YFP_RFP_NIR-422-450/495-517/566-590/710-750; Emission Range, CFP_YFP_RFP_NIR-462-484/527-551/602-680/770-850; Dichroic, CFP_YFP_RFP_NIR-459/523/598/763), an LED light source LED8 (Leica microsystems) and DIC to observe the EGFP and mCherry fluorescent signals and hyphal morphology of *A. oryzae* cells as described previously^34^.

Fluorescence images were processed by the THUNDER imaging system (Leica) with the following settings. For all observations, the inclusion agent in Instant Computational Clearing (ICC) was set to water. In addition, the Feature Scale value of the Advanced Setting in ICC was processed with the following settings: EGFP-AtuA, FITC = 0.4 µm; *btuA* mRNA, FITC = 0.4 µm; mCherry-NLS, TXRED/RFP = 1.6 µm; and mCherry-AtuA, TXRED/RFP = 0.4 µm.

### Imaging analysis

Counting of *btuA* mRNA dots was performed using the RS-FISH plugin in ImageJ Fiji^48,49^ as described previously^34^. The images of *btuA* mRNA and nuclear localization were converted from 16 to 8 bit and merged, and each hyphal region was cropped. Then, each image was re-segmented and images of nuclei were binarized and subtracted from those of *btuA* mRNA using the image calculator. The background fluorescence of the *btuA* mRNA images, in which nuclear fluorescence was eliminated, was completely subtracted by the Math function and processed with a Gaussian filter. Measurements with RS-FISH were performed with the following settings: In Radial Symmetry, Mode = Interactive, ZYX = 1.000; RANSAC was set; Use anisotropy coefficient for Dog was checked; and Spot intensity was set to Linear Interpolation. Then, *btuA* mRNA counts were measured with Adjust difference-of-gaussian values set to sigma = 1.5 and Threshold = 0.0066.

For tracking analysis, each time-lapse image was acquired for 20 s in the region of 50 µm from the hyphal tip and processed using the THUNDER imaging system described above. Then, ImageJ Fiji was used and *btuA* mRNA dynamics was analyzed using the TrackMate plug-in. The Dog Detector was used and the threshold was set to 0.3 µm to detect only intracellular-specific fluorescence. Thereafter, Lap Tracker was run with the distance between frames set to 0.8 µm, the gap distance between the three frames set to 0.8 µm and the Track displacement threshold set to 0.5 µm or greater.

### Supplementary Information

Supplementary Legends.

Supplementary Video 1.

Supplementary Video 2.

Supplementary Video 3.

Supplementary Video 4.

Supplementary Video 5.

## Data Availability

The datasets analyzed during the current study are available for *atuA* (https://fungidb.org/fungidb/app/record/gene/AO090005000840), *btuA* (https://fungidb.org/fungidb/app/record/gene/AO090009000281) and *AoapsB* (https://fungidb.org/fungidb/app/record/gene/AO090020000040).
