# Supplementary material for: Live cell imaging of β-tubulin mRNA reveals spatiotemporal expression dynamics in the filamentous fungus Aspergillus oryzae
Source: Sci Rep. 2024 Jun 14;14:13797. doi: 10.1038/s41598-024-64531-5 (PMC11178776; doi:10.1038/s41598-024-64531-5)
Supplement: Supplementary file 1 — Supplementary Legends. [file 41598_2024_64531_MOESM1_ESM.pdf]

Supplementary Information

Live cell imaging of β-tubulin mRNA reveals spatiotemporal expression dynamics in the filamentous fungus *Aspergillus oryzae*

Keishu Kawatomi[1], Yuki Morita[1], Yoshinori Katakura[1], Kaoru Takegawa[1], Adokiye Berepiki[2], Yujiro Higuchi[1,*]

[1]Department of Bioscience and Biotechnology, Faculty of Agriculture, Kyushu University, 744 Motooka, Fukuoka 819-0395, Japan

[2]FUJIFILM Diosynth Biotechnologies, Billingham, TS23 1LH, UK

*Corresponding author. Tel/Fax: +81 92 802 4734, E-mail address: y.higuchi@agr.kyushu-u.ac.jp

Word count: Abstract, 175; Main, 2818; Methods, 918.

Number of figures: 5.

Number of tables: 1.

SUPPLEMENTAL VIDEO LEGENDS

Video S1. Microtubule dynamics in the basal region.

The video of microtubule dynamics in the basal region was reconstructed from the time-lapse images shown in Fig. 1C. The strain was grown in CD medium at 30°C for 20 h. Time-lapse images are shown for 30 sec (0.50 frame/sec). Scale bar, 3 µm.

Video S2. Microtubule dynamics in the middle region.

The video of microtubule dynamics in the middle region was reconstructed from the time-lapse images shown in Fig. 1C. The strain was grown in CD medium at 30°C for 20 h. Time-lapse images are shown for 30 sec (0.50 frame/sec). Scale bar, 3 µm.

Video S3. Microtubule dynamics in the apical region.

The video of microtubule dynamics in the apical region was reconstructed from the time-lapse images shown in Fig. 1C. The strain was grown in CD medium at 30°C for 20 h. Time-lapse images are shown for 30 sec (0.50 frame/sec). Scale bar, 3 µm.

Video S4. Dynamics of microtubule-dependent *btuA* mRNA.

Co-dynamics of *btuA* mRNAs (green) and nuclei (magenta) treated with DMSO. The strain was grown in CDUU medium at 30°C for 20 h. Time-lapse images are shown for 20 sec (4.35 frames/sec). Scale bar, 10 µm.

Video S5. Dynamics of microtubule-independent *btuA* mRNA.

Co-dynamics of *btuA* mRNAs (green) and nuclei (magenta) treated with nocodazole. The strain was grown in CDUU medium at 30°C for 20 h. Time-lapse images are shown for 20 sec (4.35 frames/sec). Scale bar, 10 µm.
